# Comparison of In-Hospital Outcomes between Early and Late Catheter-Directed Thrombolysis in Acute Pulmonary Embolism: A Retrospective Observational Study

**DOI:** 10.3390/jcm13041093

**Published:** 2024-02-15

**Authors:** Majd Al Deen Alhuarrat, Kirolos Barssoum, Medhat Chowdhury, Sheetal Vasundara Mathai, Miriam Helft, Michael Grushko, Prabhjot Singh, Hani Jneid, Afaq Motiwala, Robert T. Faillace, Seth I. Sokol

**Affiliations:** 1 Division of Internal Medicine, NYC Health + Hospitals, Jacobi Medical Center, Albert Einstein College Medicine, Bronx, NY 10461, USA; alhuarrm@nychhc.org (M.A.D.A.); sheetalmathai@gmail.com (S.V.M.); robert.faillace@nychhc.org (R.T.F.); 2 Division of Cardiology, University of Texas Medical Branch, Houston, TX 77002, USA; kibarsso@utmb.edu (K.B.); hajneid@utmb.edu (H.J.); afmotiwa@utmb.edu (A.M.); 3 Ascension Providence Southfield Campus, Southfield, MI 48075, USA; medhat.chowdhury@ascension.org; 4 College of Art and Sciences, New York University, New York, NY 10003, USA; milliehelft@gmail.com; 5 Division of Cardiology, NYC Health + Hospitals, Jacobi Medical Center, Albert Einstein College of Medicine, Bronx, NY 10461, USA; michael.grushko@nychhc.org (M.G.); singhp8@nychhc.org (P.S.)

**Keywords:** acute pulmonary embolism, catheter-directed thrombolysis, outcomes, complications, management

## Abstract

The purpose of this study is to evaluate whether early initiation of catheter-directed thrombolysis (CDT) in patients presenting with acute pulmonary embolism is associated with improved in-hospital outcomes. A retrospective cohort was extracted from the 2016–2019 National Inpatient Sample database, consisting of 21,730 weighted admissions undergoing CDT acute PE. From the time of admission, the sample was divided into early (<48 h) and late interventions (>48 h). Outcomes were measured using regression analysis and propensity score matching. No significant differences in mortality, cardiac arrest, cardiogenic shock, or intracranial hemorrhage (*p* > 0.05) were found between the early and late CDT groups. Late CDT patients had a higher likelihood of receiving systemic thrombolysis (3.21 [2.18–4.74], *p* < 0.01), blood transfusion (1.84 [1.41–2.40], *p* < 0.01), intubation (1.33 [1.05–1.70], *p* = 0.02), discharge disposition to care facilities (1.32 [1.14–1.53], *p* < 0.01). and having acute kidney injury (1.42 [1.25–1.61], *p* < 0.01). Predictors of late intervention were female sex, non-white race, and weekend admission (*p* < 0.01). This study represents a comprehensive evaluation of outcomes associated with the time interval for initiating CDT, revealing reduced morbidity with early intervention. Additionally, it identifies predictors associated with delayed CDT initiation. The broader ramifications of these findings, particularly in relation to hospital resource utilization and health disparities, warrant further exploration.

## 1. Introduction

Acute pulmonary embolism (PE) remains a leading cause of death and disability in the United States. According to the updated 2024 report from the American Heart Association, there were 432,580 diagnoses of PE in the US in 2020, with the numbers of hospitalized cases showing a steady increase between 1996 and 2019 [1,2]. Additionally, the age-adjusted mortality rate for PE does not appear to have changed over a similar period [3]. The diagnosis of PE poses a challenge due to its substantial burden, often hindered by delayed recognition among both the public and medical professionals. This delay is compounded by the overlapping symptoms of PE with numerous other well-established diseases. For instance, a meta-analysis reported a 17% prevalence of PE in patients presenting with acute exacerbations of COPD [4].

Over the past decade, it has become evident that there is a paucity of knowledge about the best approach to treating acute PE due to a large data deficit. According to The American College of Chest Physicians Evidence-Based Clinical Practice (CHEST) guidelines, systemic thrombolysis is the recommended therapy for the management of hemodynamically unstable acute PE [5]. Despite this recommendation, prompt reperfusion in this clinical scenario is performed only in one out of every three acute PEs [6]. This is likely related to many absolute and relative contraindications of systemic thrombolysis, along with the gradual emergence of safer catheter-based therapies. Additionally, many acute PE cases present with stable hemodynamics, where the bleeding risk of systemic thrombolysis outweighs its potential benefits [7].

Advanced catheter-based therapy (ACBT) technology has emerged and progressed to the point where it is has become an accepted, safe, and reliable tool for the treatment of higher-risk categories of pulmonary embolism (PE) [8]. These procedures include catheter-directed thrombolysis (CDT) [9], especially ultrasound-accelerated CDT (US-CDT), the use of which is based on a few small clinical trials demonstrating favorable results in terms of reducing right ventricular (RV) strain (a marker of higher risk) and bleeding risk [10,11]. Recently, pulmonary embolism response teams (PERTs) were developed to integrate and streamline multidisciplinary teams of experts toward rapid assessment, decision making, and mobilization of resources, with the prospect of decreasing PE-associated morbidity and mortality [12]. By virtue of rapidity and expedited multidisciplinary coordination, it is plausible that a more rapid treatment intervention time, similar to the management of acute coronary syndromes, could be a way to improve outcomes. This, however, requires deeper insight into late intervention outcomes, identification of the reasons behind late intervention, and understanding of how efficacious it is to utilize such an approach.

The current body of literature exhibits a gap concerning direct trials investigating the optimal timing of CDT. Our study seeks to address this gap by investigating the association between the timing of CDT initiation and in-hospital outcomes. Utilizing a robust dataset comprising a sizable cohort of inpatients from multiple hospitals across the US, our research attempts to provide novel insights into the temporal aspects of CDT administration and the determinants influencing its timing.

## 2. Methodology

### 2.1. Database

Our research cohort of admissions with acute PE was extracted from the National Inpatient Sample (NIS) database, the largest publicly available all-payer inpatient care database in the United States. Sponsored by the Agency for Healthcare Research and Quality (AHRQ), the NIS is a part of the Healthcare Cost and Utilization Project (HCUP), which contains information on inpatient hospital admissions derived from hospital billing data. The NIS sampling frame contains, on average, 20% of the stratified sample of discharges from US hospitals, with approximately 8 million discharges per year [13]. The NIS database utilizes the International Classification of Disease, Tenth Revision, Clinical Modification and Procedure Coding System (ICD-10-CM and ICD-10-PCS), and contains data on mortality, demographics, hospital characteristics, and disposition status. Due to the publicly available nature of these data, this study was deemed to be exempt from the institutional review board at the Albert Einstein College of Medicine.

### 2.2. Sample Extraction

We queried the most recently available data from the NIS encompassing the years 2016–2019 and identified a cohort of patients with a primary discharge diagnosis of pulmonary embolism using the following ICD-10-CM codes as the primary diagnosis fields: I26.xx (except for I26.01 and I26.90) and O88.2xx (Supplementary Table S1). In order to streamline our cohort, we excluded patients who had been transferred from an outside facility and applied the following exclusion criteria: patients ≤18 years of age, missing primary outcome of mortality, concurrent diagnosis of acute ischemic stroke or acute limb ischemia (identified using appropriate codes in all diagnosis fields), and concurrent stenting or bypass procedure in the same admission (identified using appropriate codes in all the procedure fields). Secondary outcomes and complications were identified using their specific ICD codes (Supplementary Table S2). CDT alongside ultrasound-guided catheter-directed thrombolysis (US-CDT) and CDE were defined using appropriate procedure codes that have been used previously [14,15]; these codes were applied in all of the procedure fields.

### 2.3. Comorbidities Identification

Comorbidities were identified by applying ICD-10-CM codes to the secondary diagnosis fields (Supplementary Table S3), and the following were extracted: diabetes mellitus (DM), hypertension (HTN), hyperlipidemia (HLD), chronic obstructive pulmonary disease (COPD), coronary artery disease (CAD), peripheral vascular disease (PVD), heart and/or renal failure, obesity, and smoking status (active or prior). To further characterize our patient’s mortality risk from the administrative data, the Charlson’s comorbidity index (CCI) was calculated, following the ICD-10 formula by Quan et al. [16]. The sample was further characterized according to the absence of comorbidities that would significantly impact mortality (CCI = 0), or according to a low (CCI 1–2), moderate (CCI 3–4), or high risk of mortality (CCI ≥ 5).

### 2.4. Exposure and Outcomes

The exposure period was the time from admission to the procedure. Using existing NIS variables, admissions were split into those who had received CDT prior to 48 h (early intervention group) and after 48 h (late intervention group). This cutoff was prespecified following early thrombolysis trials that used 2 days as a cutoff to compare early vs. late thrombolysis therapy [17,18]. Our primary outcome was in-hospital mortality. Secondary outcomes included vasopressor use, need for concurrent blood transfusion, systemic thrombolysis, acute kidney injury, ECMO use, need for intubation, and intubation period. Similar ICD-10 codes for secondary outcomes have been used in other NIS-published research [14,15,19].

### 2.5. Statistical Analysis

Following NIS recommendations, our sample was weighted using discharge weights available in the data set. As such, the number of admissions presented in this paper was multiplied by a factor of approximately 5 to allow for data generalization. Continuous data are displayed as mean ± SD and were compared between groups using Student’s *t* test. Categorical data are displayed as frequencies and were compared using the Chi-squared test. The Jonckheere–Terpstra test was used to ascertain the significance of trends. A multivariate logistic regression model was used to calculate the odds ratio (OR) of our primary and secondary outcomes for admissions that underwent CDT after 48 h from admission in comparison to before 48 h from admission. Linear regression was adopted to compare days from admission to CDT against multiple dichotomous variables. The following co-variates were used in those regression models: age, race, sex, hospital bed size, hospital location and teaching status, CCI score, DM, HTN, HLD, CAD, COPD, PVD, heart and renal failure, obesity, and smoking history. Alongside regression analysis, and to limit confounders, we presented our results with multivariate logistic regression before and after propensity score matching. Our sample underwent propensity score matching through the greedy method, using nearest neighbor matching without replacement and a caliper of 0.05. The same variables used for the regression model were used to create our matched sample, after which unmatched observations were dropped and multivariable logistic regression was applied. The appropriateness of the matches was gauged using the standardized mean difference, and a value between −10% to 10% was considered acceptable. A *p*-value of <0.05 was considered statistically significant. All statistical analyses were performed using STATA BE, version 17.0 (StataCorp LLC., Lakeway Drive, TX, USA).

## 3. Results

**Figure 1:**
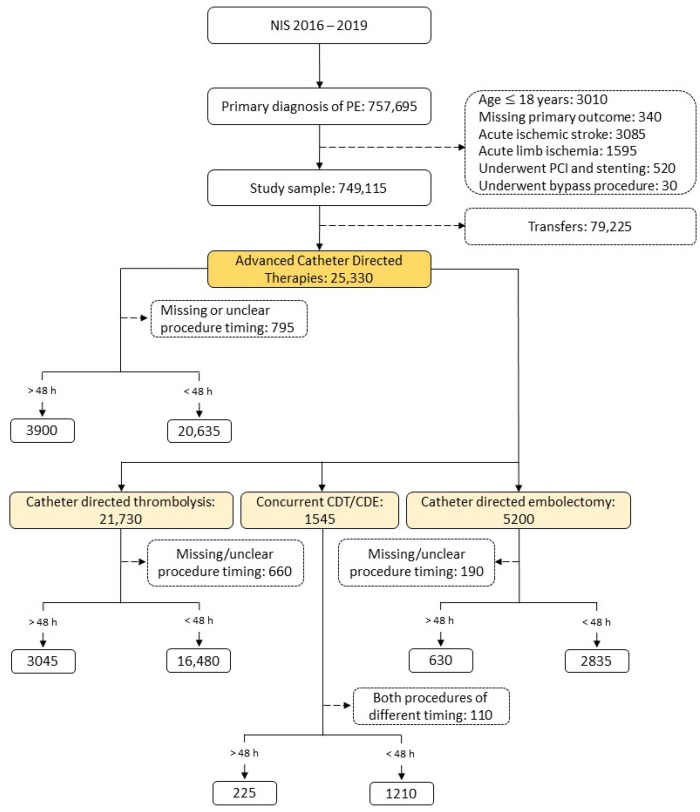
Derivation of the study sample. NIS, national in-patient sample database; CDT, catheter-directed thrombolysis; CDE catheter-directed embolectomy.

Between 2016 and 2019, there were 757,695 weighted admissions for acute PE in our cohort, which was reduced to 669,890 admissions after application of the exclusion criteria. Of these cases, 25,330 underwent ACBT. Out of those ACBTs, 24,585 patients had clear procedure timing, with 20,635 (84%) in the early intervention group and 3900 (16%) in the late intervention group. The general ACBT sample was divided into those who underwent CDT (21,730), those who underwent concurrent CDT/CDE (1545), and those who underwent CDE (5200) alone. The CDT intervention group was divided into the early (16,480) and late intervention arms (3045) (Figure 1).

Most of our population consisted of Caucasian patients (72%), and most of the procedures were performed at urban teaching hospitals (75%) (Table 1). In general, 20% of our population had COPD, 35% were either current or previous smokers, and 16% had heart failure. Prior to propensity score matching, the late intervention group had a higher CCI score (*p* < 0.01) and a higher prevalence of CAD, DM, PVD, COPD, HTN, RF, HF, HLD, and smoking (*p* < 0.01). Post-matching, all demographic variables, including CCI and comorbidities, were appropriately matched between the early and late intervention groups.

Analysis of the CDT group revealed no difference in mortality (*p* = 0.19), cardiac arrest (*p* = 0.21), cardiogenic shock (*p* = 0.17), or intracranial hemorrhage (*p* = 0.08) between the early and late treatment groups. However, there was a higher likelihood of systemic thrombolysis (aOR: 3.21, CI: 2.18–4.74, *p* < 0.01), blood transfusion (aOR: 1.84, CI: 1.41–2.40, *p* < 0.01), need for intubation (aOR: 1.33, CI: 1.05–1.70, *p* = 0.02) and prolonged intubation (24–96 h: aOR: 1.73, CI: 1.16–2.59, *p* < 0.01 and >96 h intubation: aOR: 4.65, CI: 2.59–8.34, *p* < 0.01), having acute kidney injury (aOR: 1.42, CO: 1.25–1.61, *p* < 0.01), and more likely discharge disposition to a short- or long-term care facility (aOR: 1.32, CI: 1.14–1.53, *p* < 0.01) in the late CDT intervention group (Table 2).

**Figure 2:**
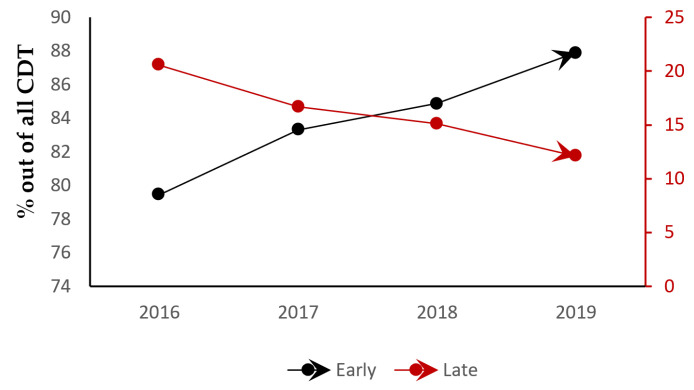
Trends in CDT (catheter-directed thrombolysis) timing across the years of the study.

Looking at a detailed breakdown of time-to-CDT (in days) against our main covariates, a higher mean time-to-CDT was noted in those who underwent ECMO, blood transfusion, systemic thrombolysis, all-cause intubation, and discharge to a care facility (*p* < 0.01) (Table 3), which is consistent with our regression analysis. Those who were admitted over the weekend were more likely to have a late CDT intervention (*beta*: 0.25, CI: 0.21–0.29, *p* < 0.01). Furthermore, through a categorical breakdown, 32% of the admissions in the late intervention arm were weekend admissions compared to 21% in the early intervention arm (*p* < 0.01). Other variables associated with late intervention in the pre-matched group were older age, female sex, non-white race, non-teaching hospitals, and hospitals with a higher bed size (*p* < 0.01) (Table 1). A linear regression analysis, however, that included the variables used in the regression for Table 3 were used to further categorize predictors of late intervention, and only female sex (*beta*: 0.12, CI: 0.09–0.16, *p* < 0.01) and non-white race (*beta*: 0.11, CI: 0.07–0.15, *p* < 0.01) were noted to be significant. A notable trend was seen toward increasing early interventions with CDT in patients being admitted for acute PE throughout our study period (*p* = 0.04) (Figure 2).

## 4. Discussion

In this analysis, there were no significant differences in the occurrence of mortality, cardiac arrest, cardiogenic shock, or intracranial hemorrhage between early and late CDT use for acute PE. On the other hand, late CDT was associated with a greater need for systemic thrombolysis, blood transfusion, intubation, ECMO, occurrence of acute kidney injury, and discharge disposition to care facilities. These findings indicate that early intervention may be advantageous in terms of reducing some of the morbidity associated with late CDT intervention. Also identified were predictors of late intervention with CDT, which included female sex, non-white ethnicity, and admissions over the weekend.

Early clinical trials of patients who received systemic thrombolysis in acute PE showed no significant difference between those who underwent systemic thrombolysis in the range of 0 to 2 days and in the range of 3 to 5 days from symptom onset [17,18,20]. However, a meta-analysis of trials of systemic thrombolysis in acute PE found that, for each day of delay from symptom onset, there was a 0.8% decrease in lung tissue perfusion and a trend toward less clot lysis [21]. More recently, a retrospective study of 456 patients treated with r-tPA for PE found that all-cause mortality, acute kidney injury, asystole, cardiogenic shock, and intubation were more common in the group that received t-PA >24–48 h from symptom onset [22].

The positive effects of CDT in acute PE have been emphasized in various studies, particularly in improving surrogate markers associated with adverse outcomes, such as right heart strain. The SEATTLE II trial studied massive and submassive PE patients treated with US-CDT [11]. It showed an improvement in the right ventricle to left ventricle dimension (RV/LV) ratio (1.55 vs. 1.13; *p* < 0.01), and pulmonary artery systolic pressure (51.4 mm Hg vs. 36.9 mm Hg; *p* < 0.01) was evident in the immediate postprocedural period. In the ULTIMA randomized control trial, the efficacy of US-CDT was compared with that of heparin alone in patients diagnosed with acute submassive PE [10]. US-CDT demonstrated superiority over heparin alone in reversing right ventricular (RV) dilation, as evidenced by a significant mean decrease in the RV/LV ratio from 0.30 at baseline to 0.03 at 24 h (*p* < 0.01). However, these studies did not investigate the timing of US-CDT from diagnosis or how it affected these parameters. The HI-PEITHO trial, which is currently enrolling, is the first randomized trial looking at hard outcomes of US-CDT and anticoagulation versus anticoagulation alone within 6 h of diagnosis; however, it does not have a comparative later-intervention group [23]. 

In a retrospective study of 41 patients undergoing US-CDT, significant improvements in the median cardiac index (0.6 L/min/ $m^{2}$ [IQR 0.4–1.1 L/min/ $m^{2}$ ] vs. 0.4 L/min/ $m^{2}$ [IQR 0.1–0.6 L/min/ $m^{2}$ ]; *p* = 0.03), median pulmonary vascular resistance (3.4 Wood units [IQR 2.5–4.1 Wood units] vs. 0.5 Wood units [IQR 9.2–1.3 Wood units]; *p* < 0.01), and mean RV stroke work index (3.5 ± 2.0 g/ $m^{2}$ /beat; *p* = 0.04) were found in the early intervention group (mean time from diagnosis: 13.3 ± 5.6 h) vs. the late intervention group (mean time from diagnosis: 46 ± 10.1 h) [24]. No significant differences in all-cause in-hospital mortality were reported. In another retrospective study published as an abstract, a total of 24 patients were enrolled and subdivided into 0–12 h, 12–24 h, and >24 h intervention post-PE diagnosis [25]. The results showed no statistical difference in the time to resolution of oxygen requirements, mortality, or bleeding, but this study was limited by its small sample size.

In our analysis, we continue to show no significant difference in mortality between the early and late CDT intervention groups; however, with our sample size, we were able to show worsened morbidity in the late group, defined as a greater need for systemic thrombolysis, blood transfusion, ECMO use, acute kidney injury, and prolonged intubation, all of which may have contributed to the notable increase in disposition to short- or long-term care facilities. The higher use of systemic thrombolysis and need for blood transfusions in the late CDT intervention group suggests that patients may have required rescue systemic thrombolysis while awaiting, or during, CDT, leading to higher bleeding rates and possibly even the need for ECMO. The confidence intervals for increased ECMO use, though, were quite wide due to the small number of patients with this requirement, making it difficult to draw any reliable conclusions.

In the realm of acute PE treatment, decisions regarding the use of CDT involve multiple considerations, with it necessitating several hours for efficacy. During the period covered by our NIS sample, CDT, particularly US-CDT, emerged as the primary new technology, while catheter-directed embolectomy (CDE) was still in its early stages, primarily used in trial settings or for high-risk patients. Patients undergoing CDE exhibited higher mortality rates, indicating its application in more severe cases [26]. The lack of mortality differences between early and late CDT interventions in our overall NIS sample, however, may stem from a lower overall risk cohort or require a longer follow-up beyond discharge to observe disparities. Future research should focus on examining long-term mortality differences post-discharge between early and late intervention groups, considering acute variations in right heart strain indices.

Disparities in healthcare, and especially in patients undergoing cardiac procedures, have been heavily described in the literature [27,28,29]. In our study, multiple patient- and admission-related factors were noted to be associated with late CDT intervention, including female sex, non-white race, and weekend admissions. Emerging studies demonstrate disparities in the presentation and severity of PE in women and certain racial/ethnic groups [30]. Administrative data from 204 hospitals in Illinois showed that non-Hispanic Black individuals had hospitalization rates for PE that were twice those of non-Hispanic Whites [31]. Black individuals were also shown to have more severe disease than Whites and to be less likely to receive intervention unless they were in the highest-risk group [32]. In another study conducted at a large urban safety net hospital, it was found that patients with government insurance had less follow-up and more readmissions for PE [33]. In a comparative study involving Hispanic and Latino patients diagnosed with PE, the findings indicated a lower likelihood of presenting with high-risk PE compared to other demographic groups, yet their in-hospital mortality rates were similar [34]. Furthermore, a subgroup analysis focusing on Hispanic and Latino individuals with higher-risk PE within the same study revealed that ethnicity did not predict the receipt of interventions or increased mortality. Future research should continue to explore these differences and complex relationships while simultaneously focusing on raising awareness regarding disparities specific to the treatment of acute PE.

Finally, the observed higher trend towards late CDT intervention for weekend admissions compared to weekdays implies uncertainty regarding the benefits of early versus later intervention in hemodynamically stable acute PE, or potentially the lack of resources available for early intervention on weekends. This underscores the significance of robust data demonstrating the advantages of early intervention in acute PE to prompt hospitals and payors toward recognizing the benefits of allocating resources and teams for optimal outcomes in this condition. Such an approach aligns with established paradigms demonstrated in other cardiovascular diseases, like acute myocardial infarction and stroke. Our study represents an initial step towards achieving this objective.

## 5. Limitations

This study is constrained by its retrospective design, which limits our capacity to establish direct causality or temporal relationships between the observed variables. Our study is also limited by being dependent on administrative data derived from coding algorithms, which are subject to bias resulting from the low sensitivity and specificity of certain ICD-10 codes; however, we mainly depended on pre-validated and already-used codes from other NIS research to minimize this bias. Furthermore, such bias affects both treatment arms equally. NIS is also devoid of lab values, imaging, and specific medication usage, limiting our ability to completely risk-stratify both treatment arms per baseline characteristic, but the use of propensity score matching helped, to an extent, in adjusting some of that unavoidable bias within the CDT subgroup. As the decision to use CDT in acute PE in real-world practice is based on proper risk stratification of patients prior to the performance of the procedure, selecting patients for inclusion based on treatment assignment may overcome some of the limitations regarding the absence of specific clinical information used to risk-stratify PE patients in the NIS database. Additionally, CDT typically requires several hours of low-dose intra-pulmonary arterial tPA, and would typically not be used in the setting of hemodynamically unstable PE, making it more likely that the studied group was in the intermediate-risk range.

## 6. Conclusions

To the best of our knowledge, this is the largest cohort comparing early versus late CDT in acute PE using a nationally representative sample from the United States. Our analysis suggests the importance of early intervention in decreasing morbidity in patients admitted with acute PE who are candidates for CDT. It also identifies predictors of delayed intervention that reveal opportunities for further research in health disparities and resource utilization, to improve outcomes related to acute PE treated with CDT and other advanced therapies. Fully understanding the relationship between time to intervention and outcomes through prospective trials and registries will help to fill the evidence gap in the modern-day approach to treating acute PE.

## Figures and Tables

**Table 1:** Baseline characteristics of patients who were admitted for a primary diagnosis of PE and underwent catheter-directed thrombolysis pre- and post-propensity score matching.

Variable		Overall	Before Propensity Matching			After Propensity Matching			Standardized Mean Difference
			Catheter-Directed Thrombolysis		*p* Value	Catheter-Directed Thrombolysis		*p* Value	
			<48 h	>48 h		<48 h	>48 h		
Population		19,525	16,480 (84%)	3045 (16%)		2970 (50%)	2970 (50%)		
Age		60.7 ± 14.6	60.4 ± 14.5	62.5 ± 14.7	<0.001	63.0 ± 14.0	62.6 ± 14.6	0.265	−0.029
Gender	Male	10,180 (52%)	8690 (53%)	1490 (49%)	<0.001	1405 (47%)	1465 (49%)	0.119	−0.040
	Female	9335 (48%)	7790 (47%)	1545 (51%)		1565 (53%)	1505 (51%)		
Race	White	13,675 (72%)	11,640 (72%)	2035 (68%)	<0.001	2045 (69%)	2030 (68%)	0.548	0.020
	Black	3795 (20%)	3155 (20%)	640 (21%)		645 (22%)	635 (21%)		
	Others	1590 (8%)	1285 (8%)	305 (10%)		280 (9%)	305 (10%)		
Hospital location and teaching status	Rural	815 (4%)	670 (4%)	145 (5%)	0.001	145 (5%)	140 (5%)	<0.001	−0.082
	Urban non-teaching	4050 (21%)	3355 (20%)	695 (23%)		545 (18%)	690 (23%)		
	Urban teaching	14,660 (75%)	12,455 (76%)	2205 (72%)		2280 (77%)	2140 (72%)		
Hospital bed size	Small	2970 (15%)	2605 (16%)	365 (12%)	<0.001	360 (12%)	360 (12%)	0.694	−0.014
	Medium	6215 (32%)	5190 (31%)	1025 (34%)		980 (33%)	1010 (34%)		
	Large	10,340 (53%)	8685 (53%)	1655 (54%)		1630 (55%)	1600 (54%)		
Charlson index	No comorbidities	7005 (36%)	6225 (38%)	780 (26%)	<0.001	725 (24%)	770 (26%)	0.395	−0.030
	Low	8655 (44%)	7175 (44%)	1480 (49%)		1490 (50%)	1455 (49%)		
	Moderate	2345 (12%)	1885 (11%)	460 (15%)		410 (14%)	425 (14%)		
	High	1520 (8%)	1195 (7%)	325 (11%)		345 (12%)	320 (11%)		
Comorbidities	Diabetes	5240 (27%)	4345 (26%)	895 (29%)	0.001	880 (30%)	860 (29%)	0.569	−0.015
	Chronic ischemic heart disease	2510 (13%)	2050 (12%)	460 (15%)	<0.001	385 (13%)	445 (15%)	0.025	0.058
	Peripheral vascular disease	740 (4%)	550 (3%)	190 (6%)	<0.001	165 (6%)	185 (6%)	0.490	0.029
	COPD	3825 (20%)	3075 (19%)	750 (25%)	<0.001	775 (26%)	745 (25%)	0.372	−0.023
	HTN	12,565 (64%)	10,520 (64%)	2045 (67%)	<0.001	1990 (67%)	2010 (68%)	0.580	0.014
	Obesity	7925 (41%)	6660 (40%)	1265 (42%)	0.243	1280 (43%)	1220 (41%)	0.115	−0.041
	Renal failure	2175 (11%)	1715 (10%)	460 (15%)	<0.001	405 (14%)	435 (15%)	0.264	0.029
	Heart failure	3120 (16%)	2510 (15%)	610 (20%)	<0.001	640 (22%)	595 (20%)	0.150	−0.037
	Smoking (current/former)	6920 (35%)	5765 (35%)	1155 (38%)	0.002	1035 (35%)	1120 (38%)	0.022	0.060
	Hyperlipidemia	7345 (38%)	6095 (37%)	1250 (41%)	<0.001	1230 (41%)	1230 (41%)	1.0	0

COPD, chronic obstructive pulmonary disease; HTN, hypertension.

**Table 2:** Odds ratios (ORs) of different in-hospital outcomes comparing early catheter-directed thrombolysis (CDT) to late CDT using multivariate logistic regression before and after propensity score matching.

Primary Outcome		Overall (%)	Catheter-Directed Thrombolysis		Multivariate Regression Pre-Match (OR [CI])	*p* Value	Multivariate Regression Post-Match (OR [CI])	*p* Value
			<48 h (%)	>48 h (%)				
Mortality		3.35	3.37	3.28	0.88 [0.71–1.10]	0.269	0.83 [0.63–1.10]	0.190
Vasopressor use		1.20	1.21	1.15	0.85 [0.59–1.23]	0.380	0.88 [0.55–1.41]	0.598
ECMO use		0.28	0.21	0.66	3.63 [1.96–6.73]	<0.001	9.03 [2.35–34.69]	0.001
Need for blood transfusion		3.15	2.76	5.25	1.73 [1.43–2.09]	<0.001	1.84 [1.41–2.40]	<0.001
Need for systemic thrombolysis		2.18	1.88	3.78	1.96 [1.57–2.45]	<0.001	3.21 [2.18–4.74]	<0.001
Acute kidney injury		19.1	17.7	26.8	1.57 [1.42–1.73]	<0.001	1.42 [1.25–1.61]	<0.001
Intracranial hemorrhage		0.46	0.46	0.49	0.97 [0.55–1.72]	0.930	2.14 [0.91–5.06]	0.081
Cardiac arrest		2.48	2.52	2.30	0.83 [0.64–1.08]	0.167	0.81 [0.59–1.13]	0.213
Cardiogenic shock		3.69	3.76	3.28	0.79 [0.64–0.99]	0.040	1.23 [0.91–1.66]	0.176
Mechanical ventilation	Overall need	4.79	4.67	5.42	1.07 [0.89–1.27]	0.469	1.33 [1.05–1.70]	0.020
	For <24 h	2.0	2.12	1.31	0.61 [0.42–0.87]	0.006	0.66 [0.42–1.02]	0.060
	For 24–96 h	2.30	2.28	2.46	1.05 [0.80–1.38]	0.728	1.73 [1.16–2.59]	0.007
	For >96 h	1.25	1.0	2.63	2.87 [2.06–4.0]	<0.001	4.65 [2.59–8.34]	<0.001
Facility discharge		13.6	12.6	18.9	1.35 [1.20–1.51]	<0.001	1.32 [1.14–1.53]	<0.001

ECMO, extracorporeal membrane oxygenation.

**Table 3:** Mean days to CDT among different variables.

	Multivariate Regression * (Linear: *Beta* [CI])	*p* Value	Days to CDT
			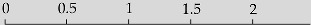
Mortality	−0.09 [−0.19–0.01]	0.08	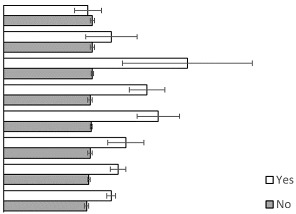
Vasopressor use	0.11 [−0.04–0.27]	0.16	
ECMO use	0.95 [0.6–1.3]	<0.01	
Blood transfusion	0.43 [0.33–0.53]	<0.01	
Systemic thrombolysis	0.58 [0.46–0.7]	<0.01	
Intubations	0.28 [0.2–0.37]	<0.01	
Facility discharges	0.19 [0.14–0.25]	<0.01	
Weekend admission	0.25 [0.21–0.29]	<0.01	

* Linear regression analysis was performed with the following variables held constant: age, race, sex, hospital location and teaching status, hospital bed size, Charlson’s index score, and multiple comorbidities, as per the initial demographics table. ECMO, extracorporeal membrane oxygenation

## Data Availability

The data underlying this article are available in the National inpatient sample database at https://www.hcup-us.ahrq.gov/ (accessed on 20 August 2023), and can be accessed by purchasing them directly from the HCUP website.

## Supplementary Materials

The following supporting information can be downloaded at: https://www.mdpi.com/article/10.3390/jcm13041093/s1, Table S1: International Classification of Diseases, 10th revision, clinical modification/procedure coding system (ICD-10 CM/PCS) codes used for sampling (inclusion and exclusion criteria); Table S2: International Classification of Diseases, 10th revision, clinical modification/procedure coding system (ICD-10 CM/PCS) codes used for the complications identification; Table S3: International Classification of Diseases, 10th revision, clinical modification/procedure coding system (ICD-10 CM/PCS) codes used for the comorbidities identification.

## Abbreviations

ACBT	advanced catheter-based therapies
aOR	adjusted odds ratio
CAD	coronary artery disease
CDE	catheter-directed embolectomy
CDT	catheter-directed thrombolysis
CCI	Charlson’s comorbidity index
COPD	chronic obstructive pulmonary disease
DM	diabetes mellitus
ECMO	extracorporeal membrane oxygenation
HLD	hyperlipidemia
HTN	hypertension
ICD	international classification of disease
PE	pulmonary embolism
PVD	peripheral vascular disease
US-CDT	ultrasound facilitated catheter-directed thrombolysis
