# Correction: Alhuarrat et al. Comparison of In-Hospital Outcomes between Early and Late Catheter-Directed Thrombolysis in Acute Pulmonary Embolism: A Retrospective Observational Study. *J. Clin. Med.* 2024, *13*, 1093

**DOI:** 10.3390/jcm13154502

**Published:** 2024-08-01

**Authors:** Majd Al Deen Alhuarrat, Kirolos Barssoum, Medhat Chowdhury, Sheetal Vasundara Mathai, Miriam Helft, Michael Grushko, Prabhjot Singh, Hani Jneid, Afaq Motiwala, Robert T. Faillace, Seth I. Sokol

**Affiliations:** 1 Division of Internal Medicine, NYC Health + Hospitals, Jacobi Medical Center, Albert Einstein College Medicine, Bronx, NY 10461, USA; alhuarrm@nychhc.org (M.A.D.A.); sheetalmathai@gmail.com (S.V.M.); robert.faillace@nychhc.org (R.T.F.); 2 Division of Cardiology, University of Texas Medical Branch, Houston, TX 77002, USA; kibarsso@utmb.edu (K.B.); hajneid@utmb.edu (H.J.); afmotiwa@utmb.edu (A.M.); 3 Ascension Providence Southfield Campus, Southfield, MI 48075, USA; medhat.chowdhury@ascension.org; 4 College of Art and Sciences, New York University, New York, NY 10003, USA; milliehelft@gmail.com; 5 Division of Cardiology, NYC Health + Hospitals, Jacobi Medical Center, Albert Einstein College of Medicine, Bronx, NY 10461, USA; michael.grushko@nychhc.org (M.G.); singhp8@nychhc.org (P.S.)

**Text correction explained:**

In the original publication [1], the conclusion of the sub-analysis, looking at predictors of late intervention, was drawn from a simple Chi-squared analysis and does not convey the true meaning of “predictors” but rather “random associations”, as it does not include the other covariates that could alter the dependent variable. Subsequently, the authors ran a multivariate regression analysis with those covariates in mind (similar to the ones used to derive the primary analysis results), and the conclusion of the sub-analysis is different as the non-significant variables were removed. As such, the authors would like to make the following corrections to the published paper:

(1) Corrections in the abstract section:

The original sentence states, “Predictors of late intervention were older age, female sex, non-white ethnicity, non-teaching hospital admission, hospitals with higher bed sizes, and weekend admission (*p* < 0.01)”.

The corrected sentence states, “Predictors of late intervention were female sex, non-white race, and weekend admission (*p* < 0.01)”.

The authors acknowledge that the primary scientific conclusions are unaffected; however, the conclusion of the secondary analysis was affected as some variables that became non-significant were removed, as above.

(2) Corrections in Results, Section 3:

The results now include the linear regression data used to derive the multivariate regression results:

The original sentence states, “Other predictors of late intervention in the pre-matched group were older age, female sex, non-white ethnicity, non-teaching hospitals, and hospitals with a higher bed size (*p* < 0.01) (Table 1)”.

The corrected sentences state, “Other variables associated with late intervention in the pre-matched group were older age, female sex, non-white race, non-teaching hospitals, and hospitals with a higher bed size (*p* < 0.01) (Table 1). A linear regression analysis, however, that included the variables used in the regression for Table 3 were used to further categorize predictors of late intervention, and only female sex (*beta*: 0.12, CI: 0.09–0.16, *p* < 0.01) and non-white race (*beta*: 0.11, CI: 0.07–0.15, *p* < 0.01) were noted to be significant”.

(3) Corrections in Discussion, Section 4:

First paragraph of the discussion:

The original sentence states, “Also identified were predictors of late intervention with CDT, which included older age, female sex, non-white ethnicity, non-teaching hospital admission, hospitals with higher bed sizes, and admissions over the weekend”.

The corrected sentence states, “Also identified were predictors of late intervention with CDT, which included female sex, non-white ethnicity, and admissions over the weekend”.

In the seventh paragraph of the discussion:

The original sentence states, “In our study, multiple patient- and hospital-related factors were noted to be associated with late CDT intervention, including older age, non-white race, female sex, non-teaching hospital status, and hospitals with higher bed sizes. Despite the low crude difference between some of these factors, the statistical difference still exists”.

The corrected sentence states, “In our study, multiple patient- and admission-related factors were noted to be associated with late CDT intervention, including female sex, non-white race, and weekend admissions”.

The original publication has also been updated.
