# Supplementary material for: Comparison of In-Hospital Outcomes between Early and Late Catheter-Directed Thrombolysis in Acute Pulmonary Embolism: A Retrospective Observational Study
Source: J Clin Med. 2024 Feb 15;13(4):1093. doi: 10.3390/jcm13041093 (PMC10889518; doi:10.3390/jcm13041093)
Supplement: Supplementary file 1 [file jcm-13-01093-s001.zip › jcm-2850924-supplementary.pdf]

Table S1

International Classification of Diseases,10th revision, clinical modification/procedure coding system (ICD-10 CM/PCS) codes used for sampling (inclusion and exclusion criteria).

Variable	ICD-10	Code(s)	
**Initial sampling** (used in primary diagnosis fields)			
Pulmonary embolism	CM	Saddle embolism	With acute core pulmonal: I26.02<br>Without acute core pulmonal: I26.92
		Other (non-saddle embolism)	With acute core pulmonal: I26.09<br>Without acute core pulmonal: I26.99
		Subsegmental PE without core pulmonal	Single: I26.93<br>Multiple: I26.94
		PE in pregnancy, labor and puerperium	O88.2, O88.211, O88.212, O88.213, O88.219, O88.22, O88.23
**Procedures** (used in all procedure fields)			
Catheter directed thrombolysis	PCS	3E06317	
Ultrasound-facilitated catheter-directed thrombolysis	PCS	6A750Z5 ,6A750Z6, 6A750Z7, 6A750ZZ, 6A751Z5, 6A751Z6, 6A751Z7, 6A751ZZ	
**Exclusion criteria** (used in all secondary diagnosis fields)			
Acute limb ischemia	CM	I743, I745, I748, I749, I7502, I75021, I75022, I75023, I75029	
Acute limb ischemia	CM	G43601, G43609, G43611, G43619, I6300, I63011, I63012, I63013, I63019, I6302, I63031, I63032, I63033, I63539, I63541, I63542, I63543, I63549, I6359, I636, I638, I6381, I6389, I639, I97810, I97811, I97820, I97821, R29700, R29701, R29702, R29703, R29704, R29705, R29706, R29707, R29708, R29709, R29710, R29711, R29712, R29713, R29714, R29715, R29716, R29717, R29718, R29719, R29720, R29721, R29722, R29723, R29724, R29725, R29726, R29727, R29728, R29729, R29730, R29731, R29732, R29733, R29734, R29735, R29736, R29737, R29738, R29739, R29740, R29741, R29742	

Revascularization	Stent & Angioplasty	PCS	Percutaneous coronary stenting:<br>“Dilation” ICD wording:<br>027034x(6,Z), 027035x(6,Z), 027036x(6,Z), 027037x(6,Z), 02703Dx(6,Z), 02703Ex(6,Z), 02703Fx(6,Z), 02703Gx(6,Z), 027134x(6,Z), 027135x(6,Z), 027136x(6,Z), 027137x(6,Z), 02713Dx(6,Z), 02713Ex(6,Z), 02713Fx(6,Z), 02713Gx(6,Z), 027234x(6,Z), 027235x(6,Z), 027236x(6,Z), 027237x(6,Z), 02723Dx(6,Z), 02723Ex(6,Z), 02723Fx(6,Z), 02723Gx(6,Z), 027334x(6,Z), 027335x(6,Z), 027336x(6,Z), 027337x(6,Z), 02733Dx(6,Z), 02733Ex(6,Z), 02733Fx(6,Z), 02733Gx(6,Z)<br>“Insertion” ICD wording (only intraluminal device; excluded insertion of “other” device):<br>02H03DZ, 02H13DZ, 02H23DZ, 02H33DZ<br>Angioplasty:<br>02703Zx(6,Z), 02713Zx(6,Z), 02723Zx(6,Z), 02733Zx(6,Z)
	CABG	PCS	021008x(3,8,9,C,F,W), 021009x(3,8,9,C,F,W), 02100Ax(3,8,9,C,F,W), 02100Jx(3,8,9,C,F,W), 02100Kx(3,8,9,C,F,W), 02100Zx(3,8,9,C,F), 02103x(44,D4), 0210444, 021048x(3,8,9,C,F,W), 021049x(3,8,9,C,F,W), 02104Ax(3,8,9,C,F,W), 02104Jx(3,8,9,C,F,W), 02104Kx(3,8,9,C,F,W), 02104Zx(3,8,9,C,F), 02104D4, 021108x(3,8,9,C,F,W), 021109x(3,8,9,C,F,W), 02110Ax(3,8,9,C,F,W), 02110Jx(3,8,9,C,F,W), 02110Kx(3,8,9,C,F,W), 02110Zx(3,8,9,C,F), 02113x(44,D4), 0210444, 021148x(3,8,9,C,F,W), 021149x(3,8,9,C,F,W), 02114Ax(3,8,9,C,F,W), 02114Jx(3,8,9,C,F,W), 02114Kx(3,8,9,C,F,W), 02114Zx(3,8,9,C,F), 02114D4, 021208x(3,8,9,C,F,W), 021209x(3,8,9,C,F,W), 02120Ax(3,8,9,C,F,W), 02120Jx(3,8,9,C,F,W), 02120Kx(3,8,9,C,F,W), 02120Zx(3,8,9,C,F), 02123x(44,D4), 0212444, 021248x(3,8,9,C,F,W), 021249x(3,8,9,C,F,W), 02124Ax(3,8,9,C,F,W), 02124Jx(3,8,9,C,F,W), 02124Kx(3,8,9,C,F,W), 02124Zx(3,8,9,C,F), 02124D4, 021308x(3,8,9,C,F,W), 021309x(3,8,9,C,F,W), 02130Ax(3,8,9,C,F,W), 02130Jx(3,8,9,C,F,W), 02130Kx(3,8,9,C,F,W), 02130Zx(3,8,9,C,F), 02133x(44,D4), 0213444, 021348x(3,8,9,C,F,W), 021349x(3,8,9,C,F,W), 02134Ax(3,8,9,C,F,W), 02134Jx(3,8,9,C,F,W), 02134Kx(3,8,9,C,F,W), 02134Zx(3,8,9,C,F), 02134D4

	Other revascularizations	PCS	027004x(6,Z), 027005x(6,Z), 027006x(6,Z), 027007x(6,Z), 02700Dx(6,Z), 02700Ex(6,Z), 02700Fx(6,Z), 02700Gx(6,Z), 02700Tx(6,Z), 02703Tx(6,Z), 027044x(6,Z), 027045x(6,Z), 027046x(6,Z), 027047x(6,Z), 02704Dx(6,Z), 02704Ex(6,Z), 02704Fx(6,Z), 02704Gx(6,Z), 02704Tx(6,Z), 027104x(6,Z), 027105x(6,Z), 027106x(6,Z), 027107x(6,Z), 02710Dx(6,Z), 02710Ex(6,Z), 02710Fx(6,Z), 02710Gx(6,Z), 02710Tx(6,Z), 02713Tx(6,Z), 027144x(6,Z), 027145x(6,Z), 027146x(6,Z), 027147x(6,Z), 02714Dx(6,Z), 02714Ex(6,Z), 02714Fx(6,Z), 02714Gx(6,Z), 02714Tx(6,Z), 027204x(6,Z), 027205x(6,Z), 027206x(6,Z), 027207x(6,Z), 02720Dx(6,Z), 02720Ex(6,Z), 02720Fx(6,Z), 02720Gx(6,Z), 02720Tx(6,Z), 02723Tx(6,Z), 027244x(6,Z), 027245x(6,Z), 027246x(6,Z), 027247x(6,Z), 02724Dx(6,Z), 02724Ex(6,Z), 02724Fx(6,Z), 02724Gx(6,Z), 02724Tx(6,Z), 027304x(6,Z), 027305x(6,Z), 027306x(6,Z), 027307x(6,Z), 02730Dx(6,Z), 02730Ex(6,Z), 02730Fx(6,Z), 02730Gx(6,Z), 02730Tx(6,Z), 02733Tx(6,Z), 027344x(6,Z), 027345x(6,Z), 027346x(6,Z), 027347x(6,Z), 02734Dx(6,Z), 02734Ex(6,Z), 02734Fx(6,Z), 02734Gx(6,Z), 02734Tx(6,Z), 02H00DZ, 02H04DZ, 02H10DZ, 02H14DZ, 02H20DZ, 02H24DZ, 02H30DZ, 02H34DZ, 02700Zx(6,Z), 02704Zx(6,Z), 02710Zx(6,Z), 02714Zx(6,Z), 02720Zx(6,Z), 02724Zx(6,Z), 02730Zx(6,Z), 02734Zx(6,Z)

Table S2

International Classification of Diseases,10th revision, clinical modification/procedure coding system (ICD-10 CM/PCS) codes used for the complications identification.

Acute kidney injury	CM	N17.xx
Vasopressors use	PCS	3E030XZ, 3E033XZ, 3E040XZ, 3E043XZ
Peripheral thrombolysis	PCS	3E03317
Blood transfusion	PCS	30230H0, 30230H1, 30230N0, 30230N1, 30230P0, 30230P1, 30233H0, 30233H1, 30233N0, 30233N1, 30233P0, 30233P1, 30240H0, 30240H1, 30240N0, 30240N1, 30240P0, 30240P1, 30243H0, 30243H1, 30243N0, 30243N1, 30243P0, 30243P1, 30250H0, 30250H1, 30250N0, 30250N1, 30250P0, 30250P1, 30253H0, 30253H1, 30253N0, 30253N1, 30253P0, 30253P1, 30260H0, 30260H1, 30260N0, 30260N1, 30260P0, 30260P1, 30263H0, 30263H1, 30263N0, 30263N1, 30263P0, 30263P1, 30273H1, 30273N1, 30273P1, 30277H1, 30277N1, 30277P1
Intracranial hemorrhage	CM	Non-traumatic subarachinoid hemorraghe: I60 (I60.0 – I60.9)<br>Non-traumatic intracerebral hemorrhage: I61 (I61.0 – I61.9)<br>Non-traumatic acute subdural hemorrhage: I62.01
Cardiac arrest	PCS	In hospital cardiopulmonary resuscitation (5A12012)<br>In hospital shock to restore cardiac rhythm (5A2204Z)
Cardiogenic shock	CM	R57.0
ECMO use	PCS	Before October 2018: 5A15223<br>After October 2018: 5A1522F, 5A1522G, 5A1522H
Intubation	PCS	Insertion of a tracheal airway: 0BH17EZ<br>Insertion of endotracheal airway into trachea: 0BH18EZ
Mechanical ventilation	PCS	5A1935Z Respiratory Ventilation, less than 24 Consecutive Hours<br>5A1945Z Respiratory Ventilation, 24-96 Consecutive Hours<br>5A1955Z Respiratory Ventilation, greater than 96 Consecutive Hours

Table S3

International Classification of Diseases,10th revision, clinical modification/procedure coding system (ICD-10 CM/PCS) codes used for the comorbidities identification.

Variable	ICD-10	Code(s)
Hypertension	CM	I10.xx + I11.xx + I12.xx + I13.xx + I15.xx + I16.xx
Diabetes	CM	E08-13.xx
Hyperlipidemia	CM	E78.xx (0-5)
Chronic ischemic heart disease	CM	I25.xx
COPD	CM	J41-47
Peripheral vascular disease	CM	I70.x, I71.x, I73.1, I73.8, I73.9, I77.1, I79.0, I79.2, K55.1, K55.8, K55.9, Z95.8, Z95.9
Smoking status	CM	F17.xx + Z72.0 + Z87.891
Heart failure	CM	I50.xx; I11.0; I13.2
Renal disease	CM	I12.0, I13.1, N03.2–N03.7, N05.2–N05.7, N18.x, N19.x, N25.0, Z49.0–Z49.2, Z94.0, Z99.2
Obesity	CM	E66.xx except E66.3
